# DNAJB3/HSP-40 Cochaperone Is Downregulated in Obese Humans and Is Restored by Physical Exercise

**DOI:** 10.1371/journal.pone.0069217

**Published:** 2013-07-24

**Authors:** Jehad Abubaker, Ali Tiss, Mohamed Abu-Farha, Fahad Al-Ghimlas, Irina Al-Khairi, Engin Baturcam, Preethi Cherian, Naser Elkum, Maha Hammad, Jeena John, Sina Kavalakatt, Abdelkrim Khadir, Samia Warsame, Said Dermime, Kazem Behbehani, Mohammed Dehbi

**Affiliations:** 1 Biomedical Research Department, Dasman Diabetes Institute, Kuwait, Kuwait; 2 Fitness and Rehabilitation Centre, Dasman Diabetes Institute, Kuwait, Kuwait; 3 Biostatistics and Epidemiology Department, Dasman Diabetes Institute, Kuwait, Kuwait; University of Pecs Medical School, Hungary

## Abstract

Obesity is a major risk factor for a myriad of disorders such as insulin resistance and diabetes. The mechanisms underlying these chronic conditions are complex but low grade inflammation and alteration of the endogenous stress defense system are well established. Previous studies indicated that impairment of HSP-25 and HSP-72 was linked to obesity, insulin resistance and diabetes in humans and animals while their induction was associated with improved clinical outcomes. In an attempt to identify additional components of the heat shock response that may be dysregulated by obesity, we used the RT^2^-Profiler PCR heat shock array, complemented with RT-PCR and validated by Western blot and immunohistochemistry. Using adipose tissue biopsies and PBMC of non-diabetic lean and obese subjects, we report the downregulation of DNAJB3 cochaperone mRNA and protein in obese that negatively correlated with percent body fat (P = 0.0001), triglycerides (*P = 0.035*) and the inflammatory chemokines IP-10 and RANTES (*P = 0.036* and *P = 0.02*, respectively). DNAJB positively correlated with maximum oxygen consumption (*P = 0.031*). Based on the beneficial effect of physical exercise, we investigated its possible impact on DNAJB3 expression and indeed, we found that exercise restored the expression of DNAJB3 in obese subjects with a concomitant decrease of phosphorylated JNK. Using cell lines, DNAJB3 protein was reduced following treatment with palmitate and tunicamycin which is suggestive of the link between the expression of DNAJB3 and the activation of the endoplasmic reticulum stress. DNAJB3 was also shown to coimmunoprecipiate with JNK and IKKβ stress kinases along with HSP-72 and thus, suggesting its potential role in modulating their activities. Taken together, these data suggest that DNAJB3 can potentially play a protective role against obesity.

## Introduction

Obesity is a medical and social problem worldwide and is a major risk factor of a myriad of health complications, particularly insulin resistance, diabetes, hypertension and cardiovascular disorders that contribute substantially to a significant reduction of both life quality and expectancy [1], [2], [3], [4]. Sedentary lifestyle and increased energy intake are known as key contributing factors to this chronic condition.

The mechanisms underlying obesity are complex but chronic-low grade inflammation and impairment of the endogenous stress defence system in key metabolic sites are considered as the main determinants that govern obesity and its associated complications [5], [6], [7], [8], [9]. Recent investigations indicated that the inflammatory and stress responses pathways are highly integrated and they work most-likely in vicious cycles, which largely explain the myriad of disorders associated with obesity [6], [10], [11]. The perturbation of the inflammatory and stress responses is known to activate several stress kinases but the best characterized are the c-Jun NH2 terminal kinase (JNK) and the inhibitor of κB kinase-β (IKKβ), both of them can phosphorylate the insulin receptor substrate-1 (IRS-1) and rendering this crucial intermediate a poor substrate for the activated insulin receptor [12], [13], [14], [15], [16].

Heat shock response (HSR); a crucial host-defense system against various pathological, physiological and environmental stressors, is one of the key pathways that was shown to be impaired in obesity-induced insulin resistance. HSR involves the immediate activation of a set of highly conserved heat shock proteins (HSPs) referred to as molecular chaperones and their master regulators; the heat shock transcription factors (HSFs) [17], [18], [19]. To maintain normal protein homeostasis (proteostasis), HSPs act with other components of the proteostasis network as a sensor to detect misfolded and aggregated proteins and through non-covalent interactions, they assist in their proper folding or targeting them to degradation through the ubiquitin-dependent proteasome and lysosome-mediated autophagy [19], [20], [21], [22]. In addition to their chaperone activity, certain HSPs have the ability to mitigate damages resulting from metabolic disorders by physically interacting with key stress and apoptotic enzymes and suppressing the inflammatory response, alleviating various forms of metabolic stress and promoting cell survival by blocking caspase-dependent apoptosis [20], [23]. HSPs can also be released into the circulation, but in contrast to their intracellular roles, extracellular HSPs exert an immune-stimulatory effect by interacting with pattern recognition receptors, such as toll-like receptors, and thereby activate the host inflammatory response [24], [25].

HSPs are broadly classified, on the basis of their apparent molecular weight, amino acid sequences and functions into distinct families. In mammalian cells, six major families of HSPs with distinct functional classes have been described to date and they consist of HSP-110/HSPH, HSP-90/HSPC, HSP-70/HSPA, HSP-60/HSPD, HSP-40/DNAJ and the small HSPs/HSPB [26], [27]. Some members of these families are ubiquitously expressed, whereas others are expressed in response to a wide variety of stress conditions and thus, highlighting their critical role in maintaining cellular homeostasis and tissue integrity [28].

The status of the HSR and its role in the pathophysiology of obesity and its complications began to be unraveled. Recent studies suggested that HSR is impaired in obesity-induced insulin resistance both in humans and experimental animal models [29], [30], [31]. The initial studies were carried out on muscle biopsies from type 2 diabetic (T2D) patients and they showed a reduction of HSP-72 expression that correlated with the degree of insulin resistance [8], [9]. These observations were further supported in experimental animal models demonstrating impaired expression of HSP-72 in the rat model of streptozotocin-induced diabetes [32] and reduced expression of both HSP-25 and HSP-72 in the insulin-resistant aged rats [52], [53]. Consistent with these findings, therapies that induce specific HSPs such as heat therapy [33], electrical therapy [34], physical exercise [35] and pharmacological drugs [36], [37], [38] are associated with beneficial outcome as monitored by improved glucose homeostasis, enhanced insulin sensitivity, reduction of visceral adiposity and suppression of the chronic inflammatory state. Taken together, these data highlight the importance of the HSR in mitigating damages associated obesity-mediated insulin resistance. A deep understanding of the status of the HSR in obese subjects prone to insulin resistance and T2D will be therefore of extreme importance.

In an attempt to identify and characterize additional components of the HSR that may be aberrantly expressed in obese subjects, we used the human RT^2^-Profiler PCR Array targeting the HSR which allows simultaneous screening of the expression profile of 84 heat shock-related genes and compared their expression pattern to control normal-weight subjects. We hypothesize that this pathway-focused approach will lead to the identification of additional genes in the HSR that may be directly linked to obesity. Using this targeted approach, we report in this study the downregulation of DNAJB3, a member of the HSP-40 in obese subject both at the RNA and protein levels. Since physical exercise is known to modulate the stress response, to reduce inflammation and to improve insulin signaling, we investigated its possible effect on the expression of DNAJB3. We report here for the first time that physical exercise increased the expression of DNAJB3 in a manner that was concomitant with decreased phosphorylation of JNK in obese subjects. Using cell lines, the reduction of DNAJB3 protein was linked to the activation of the endoplasmic reticulum stress and in coimmunoprecipitation assays; DNAJB3 was part of a complex containing HSP-72 along with JNK and IKKβ stress kinases. Taken together, our data support the protective role that DNAJB3 may play against obesity.

## Materials and Methods

### Study population

The study was conducted on adult male and female subjects consisting of lean (BMI = 20–24.9 kg/m^2^) and obese (BMI = 30–40 kg/m^2^). Informed written consent was obtained from all subjects before their participation in the study which was approved by the Review Board of Dasman Diabetes Institute and carried out in line with the guideline ethical declaration of Helsinki. Participants that followed any physical exercise within the last 6 months prior to this study, morbid obese (i.e. BMI >40 kg/m^2^) and participants with prior major illness or intake of medications and/or supplements known to influence the body composition, bone mass were excluded from the study. The physical characteristics of the participating subjects are shown in Table 1.

**Table 1:** Physical characteristics of subjects at baseline.

	Lean (n = 54)	Obese (n = 66)	*P-value*
**Age (year)**	37.24±10.89	44.88±12.12	*0.0004*
**Gender (Males/ Females)**	18/36	37/29	*0.013*
**BMI (kg/m2)**	22.39±2.09	34.57±2.95	*<0.0001*
**PBF (%)**	27.37±5.13	38.37±5.01	*<0.0001*
**Waist (cm)**	80.74±15.84	108.53±12.16	*<0.0001*
**Hip (cm)**	92.29±14.67	118.55±8.27	*<0.0001*
**Waist/Hip**	0.86±0.10	0.92±0.10	*0.003*

*Data are presented as mean ± SD. BMI (body mass index), PBF (percent body fat).*

### Exercise Protocol

All eligible subjects were enrolled to a supervised exercise program at the Fitness and Rehabilitation Center (FRC) of Dasman Diabetes Institute. Prior to exercise prescription, each individual underwent a symptom-limited maximal incremental cardiopulmonary exercise test “CPET” (COSMED Quark, Italy) using an electromagnetically braked cycle ergometer. The CPET was primarily used to determine the maximum heart rate (max HR) as well as the response to aerobic exercise as measured by the maximum oxygen consumption (V_O2 Max_) for each subject. Thereafter, a physical fitness assessment test was performed to determine muscle strength and endurance along with flexibility by performing grip strength (dynamometer), push-ups (upper body strength), sit-ups and forward bending test (both upper and lower body flexibility). The exercise training involves a combination of both moderate intensity of aerobic exercise and resistance training using either treadmill or cycling. Each exercise session includes 10 minutes warming-up and cooling down steps at 50–60% of max HR, along with 40 minutes of the prescribed exercise program at 65–80% of max HR. For the duration of the 3-months period, participants exercised 3 to 5 times per week and they were instructed to reach and maintain the recommended heart rate range. This was achieved by regular monitoring of the heart rate during the aerobic training. Strength training was performed 2 to 3 times a week according to the program plan. Exercise intensity, duration and blood pressures were recorded for each session. All trainings were supervised by qualified fitness professionals from FRC. To assess the effectiveness of the exercise, the same physical stress and fitness tests were performed for all subjects at the end of the exercise program.

### Blood and tissue sampling

Venous peripheral blood and subcutaneous adipose tissue biopsies were obtained before starting the exercise (baseline) and after 3-months of exercise. Peripheral blood mononuclear cells (PBMCs) were prepared from blood using Ficoll-Hypaque density gradient centrifugation method. Plasma and serum were prepared using vacutainer tubes and then aliquoted and stored at −80°C. Subcutaneous superficial adipose tissue biopsies (∼1 g) were obtained from the periumbilical area by surgical biopsy after a local anesthesia. Once removed, the biopsy was rinsed in cold PBS, divided into 4 pieces and stored appropriately until assayed.

### Anthropometric measurements and blood biochemistry

Anthropometric measurements were taken at the baseline and after 3-months of exercise. Whole-body composition was determined by dual-energy radiographic absorptiometry device (Lunar DPX, Lunar radiation, Madison, WI). Glucose and lipid profiles were measured on the Siemens Dimension RXL chemistry analyzer (Diamond Diagnostics, Holliston, MA). HbA1c was determined using the Variant^TM^ device (BioRad, Hercules, CA). Plasma levels of inflammatory and metabolic markers were measured using bead-based multiplexing technology. Median fluorescence intensities were collected on a Bioplex-200 system using Bio-plex Manager software version 6 (BioRad, Hercules, CA). Lipid peroxidation was assessed by measuring plasma levels of malonaldehyde, using TBARs Assay Kit (Cayman Chemical Company, Ann Arbor, MI). Serum levels of ROS were determined using the OxiSelect™ ROS Assay Kit (Cell Biolabs Inc, San Diego, CA). All the above assays were carried out according to the instructions of the manufacturers.

### RNA extraction and Reverse Transcription

Total RNA was extracted from PBMC using AllPrep RNA/Protein Kit (Qiagen, Inc., Valencia, CA) and adipose tissue using The RNeasy Lipid Tissue Mini Kit (Qiagen, Inc., Valencia, CA). Quantity and quality of the RNA were determined using the Epoch spectrophotometer system (BioTek Instruments, Inc., Winooski, VT). 1 μg of each RNA sample was reverse-transcribed to cDNA using High Capacity cDNA Reverse Transcription Kits (Applied Biosystems, Foster City, CA) or RT^2^ First Strand Kit (SABioscience/Qiagen, Valencia, CA). All the procedures were carried out according to the manufacturer's instructions.

### Measurement of gene expression by Real-time Quantitative PCR

Human Heat Shock Protein RT^2^-profiler PCR Arrays (SABiosciences/Qiagen, Inc., Valencia, CA) is a quantitative SYBR Green-based real-time PCR for analyzing the expression of focused panel of genes simultaneously. It contains 84 specific cDNA fragments of heat shock related genes, plus five housekeeping genes for normalization consisting of beta-2-microglobulin, ribosomal protein L13a, hypoxanthine guanine phosphoribosyl transferase 1, glyceraldehyde-3-phosphate dehydrogenase and actin beta. For each array, 200 ng of the template cDNAs were mixed with RT^2^ Real time SYBR Green qPCR master mix. Equal aliquots of this mixture were loaded to each well containing pre-dispensed gene-specific primer sets as recommended by the manufacturer and run on the Rotor-Disc 100 system (Qiagen, Inc., Valencia, CA). Rotor-Gene Q version 2 software was used to calculate the cycle threshold (CT) values for all the genes on each PCR array (Qiagen, Inc., Valencia, CA). Each replicate CT was normalized to the average CT of 5 reference genes present in each run. The following formula was used to calculate the relative amount of transcripts in the obese and lean groups after normalization with the five internal controls: ΔΔCT  =  ΔCT (obese group)-ΔCT (control group) for RNA samples as described elsewhere [67]. Changes in gene expression between the two groups were determined using a 2-tailed t-test and the difference was presented as a fold increase/decrease. Only genes showing more than 1.5-fold change with a P-value less than 0.05 were retained. Genes showing differential expression were further validated by conventional quantitative real time PCR using primers corresponding to the genes of interest.

### Cell Culture, plasmids and transfections

Human embryonic kidney (HEK-293), human acute monocytic leukemia (THP1) and L6 rat skeletal muscle cell lines were obtained from American Type Culture Collection (Rockville, Baltimore, MD). They were cultured in Eagle's Minimum Essential Medium (EMEM) supplemented with 10% fetal bovine serum and penicillin/streptomycin. Stimulation of cells with inflammatory cytokines (R & D Systems, Minneapolis, MN), H_2_O_2_, palmitate and tunicamycin (Sigma Aldrich, St. Louis, MO) was done for overnight. The plasmids used in this study consisted of pCMV-DNAJB3 (OriGene Technologies, Inc., Rockville, MD) which encodes for human DNAJB3 was cloned as an NH_2_-terminal fusion with Myc-FLAG tag. pCMV-ATF-6 (a kind gift from Dr. Ron Prywes, Dept. Biological Sciences, Columbia University, New York, USA) encodes for ATF-6 protein as an NH_2_-terminal fusion with FLAG tag. pcDNA3.1 (Invitrogen, Carlsbad, CA) was used as a control empty vector. For transient transfection assays, HEK-293T cells (at ∼80% of confluence) were transfected with 20 μg of DNA using Lipofectamine method as recommended by the manufacturer (Invitrogen, Carlsbad, CA). Following transfection, cells were incubated in complete EMEM media for 48 hours and then, harvested for coimmunoprecipitation experiments.

### Coimmunoprecipitations

Approximately 2×10^7^ of HEK-293 transfected cells were washed twice with ice-cold PBS and lysed in 1 ml of lysis buffer (50 mM Tris-HCl, pH 7.4, 150 mM NaCl, 1 mM EDTA and 1% Triton X-100) containing a cocktail of protease inhibitors Mini Complete (Roche Diagnostics, Laval, Quebec) for 30 min at 4°C. Extracts were centrifuged at 14,000 rpm for 10 minutes at 4°C to remove cell debris. 500 μg of total cell lysates were added to 100 μl of a 50% slurry of anti-FLAG M2 affinity agarose beads (Sigma Aldrich, St. Louis, MO), preequilibrated with cold washing buffer (50 mM Tris-HCl pH 7.4 and 150 mM NaCl) and incubated overnight at 4°C with continuous end-over-end rotation. Protein complex was collected by centrifugation and washed four times in washing buffer and bound proteins were eluted with 100 μl of 3xFLAG tag peptide at 150 μg/ml as recommended by the manufacturer (Sigma Aldrich, St. Louis, MO). To detect the endogenous formation of DNAJB3/JNK/HSP-72 complex, HEK293 cells were lysed as described above and 500 μg of total cell lysates were used in immunoprecipitation assays using either anti-DNAJB3 or anti-HSP-72 antibodies for pull down. Eluted samples were then fractionated on SDS-PAGE and transferred to PVDF membranes for immunoblotting as described below.

### Western blot analysis

Western blots were carried out on whole PBMC extracts or cell extracts prepared in RIPA buffer (50 mM Tris-HCl pH7.5, 150 mM NaCl, 1% Triton ×100, 1 mM EDTA, 0.5% Sodium deoxycholate and 0.1% SDS). Protein concentration was determined by Bradford method using globulin as a standard and 20 μg of proteins were resolved on 10% SDS-PAGE gels. Proteins were then transferred onto PVDF membranes, blocked with 5% non-fat dried milk in Tris-buffered saline containing 0.05% Tween 20 (TBST) for 1 h at room temperature (RT) and then probed with the primary antibody for overnight at 4°C. After washing, the membranes were incubated with horseradish peroxidase-conjugated secondary antibody for 2 h at RT and finally, protein bands were visualized by chemiluminescence and the images were captured by using the Versadoc 5000 system (BioRad, Hercules, CA). The primary antibodies used in this study are raised against DNAJB3 (Proteintech Group, Inc., Chicago, IL), DNAJC5B (Abcam, Cambridge, MA), DNAJB7 (Atlas Antibodies, Stockholm, Sweden). The remaining anti-HSPs antibodies were purchased from Enzo Life Sciences (Life Sciences International, Inc. Plymouth Meeting, PA) and they are raised against HSP-60, the constitutive HSP-70 (HSC-70), the inducible HSP-70 (HSP-72) and HSP-90. Antibodies against total JNK, Phospho-JNK and IKKβ were purchased from Cell Signaling (Cell Signaling Technology,Inc., Danvers, MA). Actin (Santa Cruz Biotechnology, Santa Cruz, CA) and GAPDH (Millipore, Temecula, CA) were used as internal controls. For densitometric analysis, the intensity of the bands was determined using Quantity One Software (BioRad, Hercules, CA).

### Immunohistochemistry

Formalin fixed, paraffin embedded adipose tissue samples were prepared and used to make sections for immunohistochemical studies as described previously [68]. Briefly, sections were deparaffinized and the antigens were retrieved at high-temperature using antigen unmasking solution (Dako, Denmark). The endogenous peroxidase was quenched using 3% H_2_O_2_ (Merck Schuchardt, Gemany) for 60 min at RT. Sections were blocked with 5% fat-free milk for 60 min at RT followed by 1% BSA for another 60 min and then, incubated at 4°C for overnight with primary antibodies. After washing, sections were stained with horseradish conjugated secondary antibody (Dako, Denmark) for 60 minutes at RT. Colors were developed using DAB kit (Dako, Denmark) and sections were counterstained with hematoxylin (Sigma Aldrich, St. Louis, MO). Quantification of the immunohistochemical staining data was done using Aperio software version 6.3 (Molecular Devices, Downingtown, PA) with an established arbitrary threshold.

### Statistical analysis

Statistical analyses were performed with SAS version 9.2 (SAS Institute Inc, Cary, NC). Unless otherwise stated, all descriptive statistics for the variables in the study were reported as means ± standard deviation (SD). Student's t-test was used to determine significance of difference in means between the two groups. Correlations between variables were calculated with the Spearman's rank correlation test. Differences were considered statistically significant at *P*-values less than 0.05.

## Results

### Baseline characteristics of study population

The physical characteristics of the two groups enrolled in this study are displayed in Table 1. As expected, body mass index (BMI), percent body fat (PBF), and waist and hip circumferences were significantly higher in obese group compared to lean group (*P<0.0001*). Given that age and gender were significantly different between the two groups, we adjusted the clinical and metabolic parameters displayed in Table 2 accordingly. Obese subjects had higher systolic blood pressure (SBP; *P = 0.01*) and lower capacity of maximal oxygen uptake (V_02 Max_; *P = 0.03*) as shown in Table 2. They also had decreased levels of HDL and increased levels of TG (*P<0.0001* and *P = 0.0002*, respectively). Compared to lean group, the metabolic profile is dysregulated in obese subjects as indicated by increased levels of C-peptide (*P* = *0.03*), glucagon (*P* = *0.048*), leptin (*P<0.0001*) and PAI-1 (*P = 0.013*). Likewise, obese subjects displayed higher inflammatory response as measured by IP-10 and RANTES chemokines (*P* = *0.003* and *P = 0.012*, respectively), but they did not differ significantly in the rest of the inflammatory mediators as well as the oxidative stress markers (Table 2 and data not shown).

**Table 2:** Clinical and biochemical characteristics of subjects at baseline.

	Lean (n = 54)	Obese (n = 66)	*P-value*
**Resting HR (beat/min)**	80.71±14.51	77.43±8.15	*0.73*
**SBP (mmHg)**	113.00±10.81	127.50±11.89	*0.01*
**DBP (mmHg)**	76.43±6.33	82.00±10.14	*0.13*
**V_O2, max_ (ml/kg/min)**	21.63±3.76	17.48±4.83	*0.03*
**Cholesterol (mmol/l)**	5.03±0.92	5.26±1.02	*0.31*
**HDL (mmol/l)**	1.43±0.49	1.13±0.24	*<0.0001*
**LDL (mmol/l)**	3.13±0.85	3.43±0.96	*0.14*
**TG (mmol/l)**	0.91±0.43	1.52±0.91	*0.0002*
**Glucose (mmol/l)**	5.02±0.64	5.45±0.92	*0.17*
**HBA1C (%)**	5.47±0.40	5.93±1.03	*0.056*
**C-peptide (ng/ml)**	2.44±0.71	3.68±2.31	*0.03*
**Glucagon (ng/ml)**	0.65±0.11	0.71±0.15	*0.048*
**GLP-1 (ng/ml)**	2.62±0.83	2.68±1.54	*0.68*
**Insulin (ng/ml)**	2.35±1.12	4.51±2.11	*0.12*
**Leptin (ng/ml)**	4.82±3.03	9.72±6.91	*<0.0001*
**PAI-1 (ng/ml)**	3.19±1.55	4.14±1.53	*0.013*
**TNF-α (pg/ml)**	23.70±9.30	25.70±13.20	*0.53*
**IL-1β (pg/ml)**	1.20±0.47	1.35±0.85	*0.83*
**IL-6 (pg/ml)**	4.99±2.18	4.68±1.87	*0.54*
**IL-10 (pg/ml)**	1.97±1.39	2.33±2.24	*0.43*
**IP-10 (ng/ml)**	0.39±0.14	0.60±0.32	*0.003*
**RANTES (ng/ml)**	1.29±0.60	1.78±0.86	*0.012*
**ROS (mM)**	1.38±0.37	1.58±0.63	*0.24*
**TBARS (μM)**	1.29±0.48	1.52±0.43	*0.11*

*Data were adjusted for age and gender and presented as mean ± SD. HR (heart rate), SBP (systolic blood pressure), DBP (diastolic blood pressure), V_O2 Max_ (maximum oxygen consumption), HDL (high density lipoprotein), LDL (low density lipoprotein) and TG (triglycerides).*

### RT^2^ Profiler PCR Array for Hsp-related genes

Despite the central role that HSPs play against a variety of diseases, only a few studies that documented their role in obesity, insulin resistance and diabetes. In addition, these studies were primarily focused on HSP-25, HSP-72 and to a lesser extent; HSP-60 [8], [9], [33], [39]. Therefore, we decided to perform an expression profiling analysis on a selected set of Hsp-related genes using RT^2^-Profiler PCR heat shock array. RNA samples prepared from PBMC of 6 non-diabetic subjects divided into lean and obese (n = 3 for each group). Among the 84 Hsp-related genes screened by this approach and after normalization with the reference genes, only seven genes showed differential expression between the two groups in which, five genes were up-regulated and two genes were down-regulated in obese subjects (Data not shown). Members of the *Hsp*-40 family, showing a downregulation in obese participants are the subject of the current investigation.

### Reduced expression of members of the *Hsp*-40 family in PBMC and adipose tissue of obese subjects

**Figure 1:**
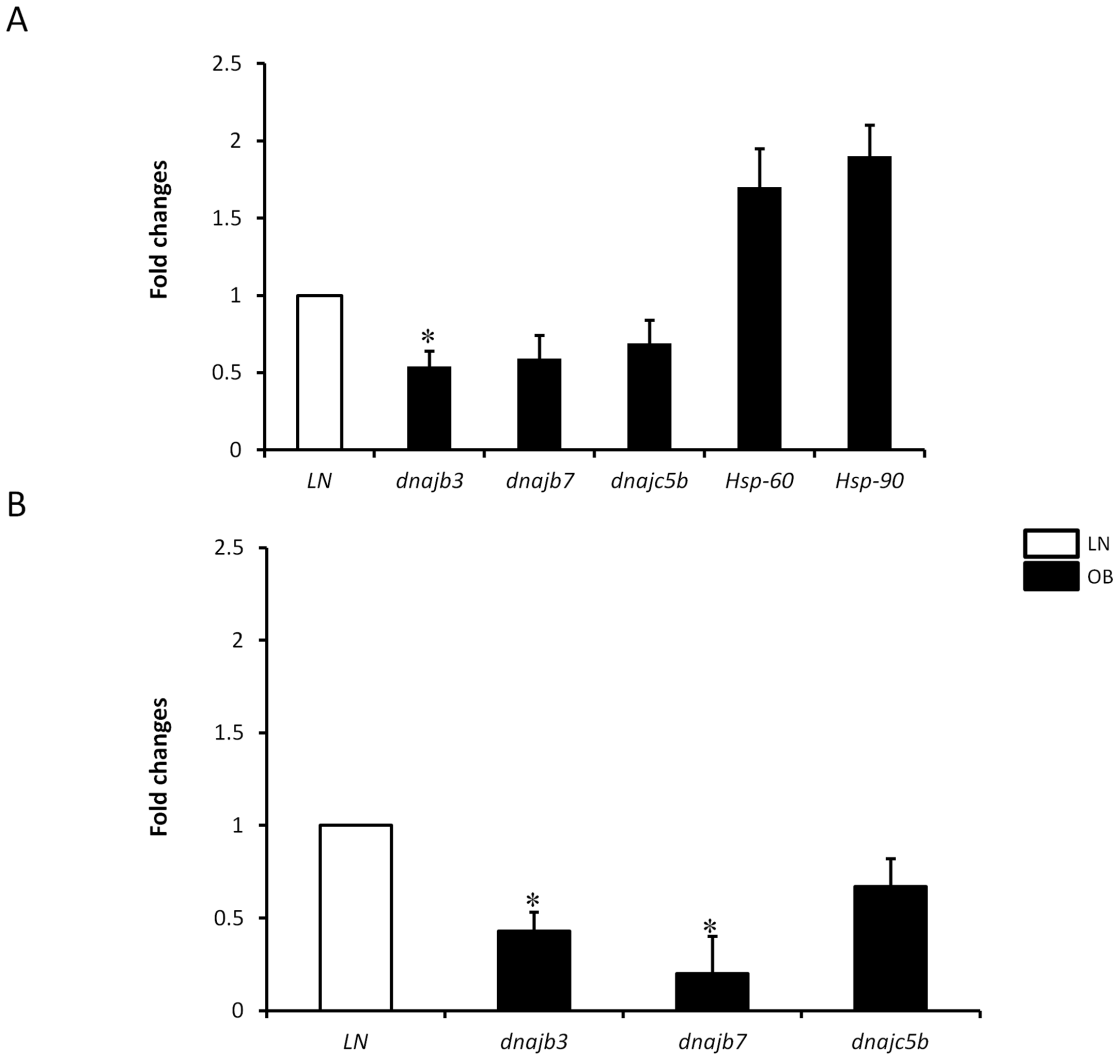
Downregulation of members of *Hsp*-40 in obese subjects. Total RNA was isolated from PBMC (A) and adipose tissue biopsies (B) of lean (n = 14) and obese (n = 17) non-diabetic participants and subjected to quantitative analysis using real-time PCR. The data are presented as fold changes in obese compared to lean subjects. * *P*<0.05 as determined using student's t-test.

To validate the RT^2^-Profiler PCR array data, we performed real-time PCR analysis on individual genes using RNA from PBMC of 10 additional non-diabetic obese and lean subjects (n = 5 for each group). We investigated the expression profile *dnajc5b* and *dnajb7* that were identified in the initial RT^2^-Profiler screening array, but subsequently, we included other heat shock-related genes, including *dnajb*3 gene that were not represented in the RT^2^-profiler heat shock array (Table 3). The inclusion of *dnajb*3 was based on a recent descriptive gene expression profiling study showing a downregulation of *dnajb*3 mRNA in obese mice compared to lean mice [40]. Consistent with the RT^2^-Profiler PCR array data, *dnajc5b* and *dnajb7* showed more than 1.5-fold decrease in obese compared to lean group (Fig. 1A). Likewise, the expression of *dnjab3* was also significantly reduced in obese subjects (*P = 0.037*). In contrast, *Hsp-60* and *Hsp-90* expression was increased by more than 1.5-fold in obese subjects, albeit this increase was not statistically significant (Fig. 1A). No change has been found in the expression of other Hsp-related genes (data not shown). Given the central role played by the adipose tissue in the pathophysiology of obesity, we next investigated whether obesity triggers a reduction in the expression levels of *dnaj* genes in this dynamic organ. Using real time PCR analysis, we found a more pronounced reduction in the expression of *dnajb3* (2.3-fold; *P = 0.026*), *dnajb7* (4-fold; *P = 0.04*) and *dnajc5b* (1.7-fold) in obese subjects (Fig. 1B). Taken together, these results indicate that obesity is associated with a significant reduction in the expression of the 3 members of *Hsp-40* both in PBMC and adipose tissue.

**Table 3:** Primer sequences used for real time PCR to analyze gene expression status of human heat shock-related genes.

Genes	Primers
*dnajb3*	Forward: 5'-ATCCGAGGCCATCAAGAAG-3' Reverse: 5'-CCACCTGCTTGAATCTCCTC-3'
*dnajb7*	Forward: 5'-CGGAGGTGGAAGTCATTTTG-3' Reverse: 5'-AGGAGCTTCCTGGACGATTT-3'
*dnajc5b*	Forward: 5'-ACGGTGGAACAGTTTTGCAGC-3' Reverse: 5'-TTTCTTCATTTGATGCTCCCTTA-3'
*Hsp-60*	Forward: 5'-GATGTCCTGGGCTGTTTCAT-3' Reverse: 5'-GCCTCGATCAAACTTCATGC-3'
*Hsp-90*	Forward: 5'-ACTTAGCCAAGATGCCTGAGG-3' Reverse: 5'-CACCCCCAAGAAGTTCACAC-3'
*hspe1*	Forward: 5'-GTGCAGTGGAGGGAAAAGAA-3' Reverse: 5'-CGGCCTATTGAGGACAATTT-3'
*hspa14*	Forward: 5'-GTGCAGTGGAGGGAAAAGAA-3' Reverse: 5'-CGGCCTATTGAGGACAATTT-3'
*GAPDH*	Forward: 5'-AGGGCTGCTTTTAACTCTGGT-3' Reverse: 5'-CCCCACTTGATTTTGGAGGGA-3'

### Validation of the transcriptomic data by Western Blotting and Immunohistochemistry

**Figure 2:**
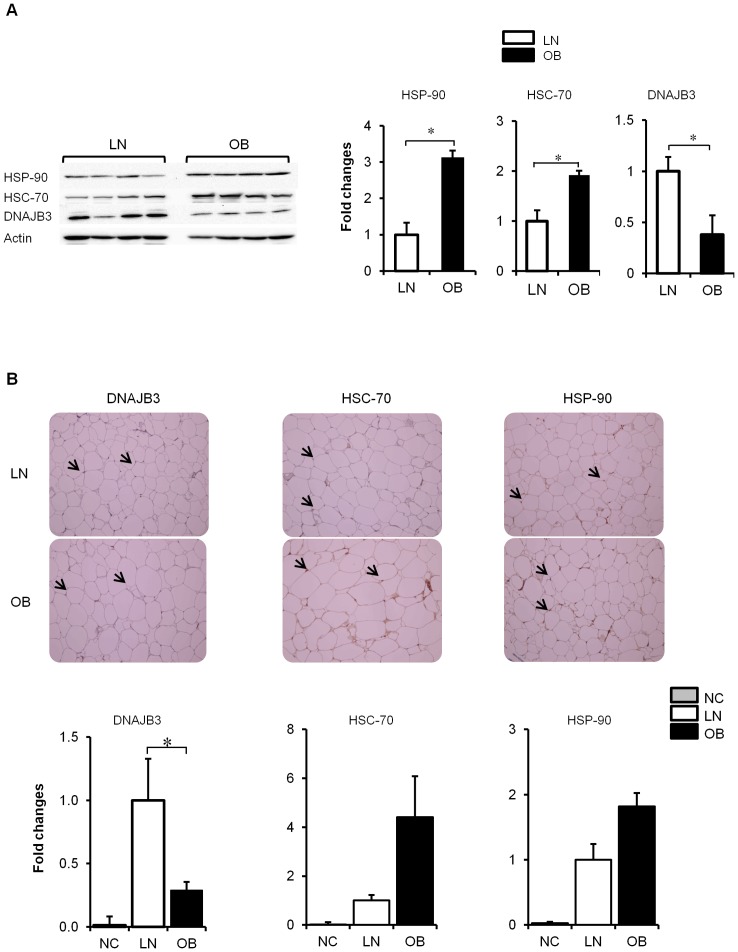
Obesity triggers a downregulation of DNAJB3 protein. (A) Total proteins were extracted from PBMC of lean (n = 4) and obese (n = 4) non-diabetic participants and subjected to western blot using the indicated antibodies. The bands were quantified as described in materials and methods and the relative intensity was determined after correction with actin that was used as internal control to monitor loading efficiency. The data are presented at the bottom as fold changes compared to lean group. The blots shown are representative of at least three independent experiments with consistent results. (B) Immunohistochemical staining using subcutaneous adipose biopsies from lean (n = 4) and obese (n = 11) non-diabetic participants. Aperio software was used to quantify positive staining (indicated by arrows) and the values are illustrated at the bottom as fold changes compared to lean. As negative control (NC) for the experiment, the primary antibodies were omitted. * *P*<0.05 as determined using student's t-test.

In order to validate the RT-PCR data on these members of the *Hsp*-40 at the protein level, we performed Western blot analysis on PBMCs and immunohistochemical (IHC) analysis on adipose tissue from selected subjects. As shown in Figure 2A, Western blot analysis performed on lean and obese subjects (n = 4 for each group) showed a major reduction in the expression of DNAJB3 protein in obese subjects (*P<0.05*). Under the same conditions, we were unable to validate the expression of DNAJC5B and DNAJB7 (data not shown). In contrast to DNAJB3, the expression levels of HSC-70 and HSP-90 were increased in obese subjects (Fig. 2A). Consistent with Western blot analysis, IHC studies on adipose tissue biopsies isolated from lean (n = 4) and obese (n = 11) subjects indicated a significant reduction of DNAJB3 (*P<0.05*) and an increase in the expression of HSP*-*70 and HSP-90 (Fig. 2B). Hence both Western blot and IHC data were in complete agreement with each other and they fully support the gene expression data obtained on *dnajb3*, *Hsp-70* and *Hsp-90*.

### Effect of physical exercise on DNAJB3 expression

**Figure 3:**
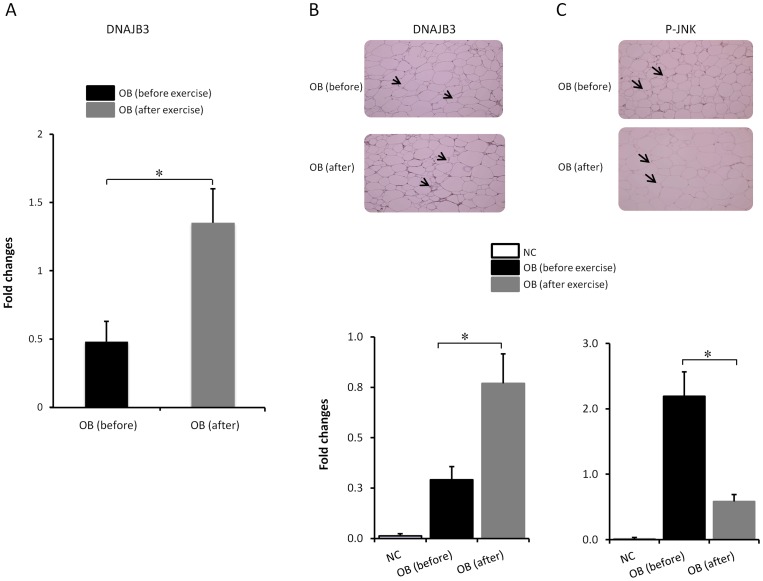
Physical exercise restores the expression of DNAJB3. (A) Quantitative analysis of DNAJB3 mRNA levels in the adipose tissue from obese before exercise (n = 10) and after 3 months of exercise (n = 10) using real-time PCR. (B and C) Immunohistochemical staining using subcutaneous adipose biopsies from obese subjects before exercise (n = 11) and after 3 months of exercise (n = 7) using DNAJB3 (B) and Phopsho-JNK (C) antibodies. Arrows indicate the positive staining. Aperio software was used for quantification and the values are illustrated at the bottom as fold changes after exercise. Student's *t-test* for two group analysis was done to compare the expression of DNAJB3 (B) and JNK (C) in obese before and after exercise. *: *P*<0.05.

The beneficial effect of physical exercise on weight loss and improving clinical manifestations associated with obesity prompted us to test if it has an effect on the expression of DNAJB3 and if so, whether these levels correlated with the post-exercise training data on physical and metabolic parameters. To investigate the effectiveness of the exercise protocol assigned for participants, we performed a pairwise comparison of physical, clinical and metabolic parameters in obese subjects (n = 24) before and after physical exercise. Table 4 shows that although there was no significant change in the BMI, waist and hip after 3 months of exercise, there was a significant reduction of PBF and SBP (*P = 0.023* and *P = 0.01*, respectively) and increase in V_O2 Max_ (*P = 0.01*) along with reduced inflammatory response as indicated by reduced levels of TNF-α and IL-6 (*P = 0.004* and *P = 0.012*, respectively). Exercise triggered also a decrease in TBARS levels (*P = 0.004*). In order to assess the effect of exercise on the expression of DNAJB3, fat adipose tissue biopsies were collected from obese subjects that completed the exercise program and used to monitor the expression of DNAJB3 (n = 10). As shown in Figure 3A, there was a significant increase in the levels of DNAJB3 mRNA in obese subjects after exercise (*P = 0.005*). Consistent with the RT-PCR data, IHC analysis confirmed that DNAJB3 protein is significantly increased in the adipose tissue of obese subjects after physical exercise (Fig. 3B; *P = 0.003*). Taken together, these data suggest that exercise can interfere with obesity-mediated repression of DNAJB3. Since obesity is known to trigger the activation of the stress kinase JNK; particularly its phosphorylation, and with the perspective to establish a correlation between the levels of DNAJB3 and activated JNK, we measured the levels of phosphorylated JNK in adipose tissue by IHC before and after exercise. In contrast to the pattern observed for DNAJB3, there was a significant increase in the levels phosphorylated JNK in obese subjects compared to lean subjects (*P = 0.016,* data not shown). Furthermore, the expected increase in phosphorylated JNK in obese was significantly reduced by physical exercise (Fig. 3C; *P = 0.0013*). No effect was observed for total JNK before and after exercise (data not shown). From this experiment we concluded that there was an inverse correlation between DNAJB3 and activated JNK.

**Table 4:** Physical and clinical characteristics of 24 obese subjects before and after exercise.

	Before exercise	After exercise	*P-value*
**Age (year)**	47.78±13.02	–	–
**Gender (M/F)**	17/7	–	–
**BMI (kg/m^2^)**	34.60±2.95	33.93±2.44	*0.18*
**PBF (%)**	37.48±5.35	36.63±5.71	*0.022*
**Waist (cm)**	108.93±11.32	107.33±9.10	*0.102*
**Hip (cm)**	117.51±9.42	117.88±6.59	*0.19*
**Resting HR (beat/min)**	77.43±8.15	77.00±10.30	*0.89*
**SBP (mmHg)**	127.50±11.89	119.29±8.29	*0.01*
**DBP (mmHg)**	82.00±10.14	77.86±4.26	*0.11*
**V_O2 Max_ (ml/kg/min)**	17.48±4.83	19.66±5.17	*0.01*
**Cholesterol (mmol/l)**	5.26±1.03	5.18±0.99	*0.28*
**HDL (mmol/l)**	1.16±0.25	1.05±0.25	*0.073*
**LDL (mmol/l)**	3.35±0.95	3.40±0.94	*0.72*
**TG (mmol/l)**	1.71±0.93	1.63±0.71	*0.76*
**Glucose (mmol/l)**	5.76±1.08	5.91±1.03	*0.84*
**HbA1C (%)**	6.17±1.44	5.99±0.56	*0.46*
**C-peptide (ng/ml)**	2.89±1.48	3.40±1.16	*0.11*
**Glucagon (ng/ml)**	0.63±0.16	0.54±0.10	*0.056*
**GLP-1 (ng/ml)**	2.31±1.66	1.83±0.45	*0.052*
**Insulin (ng/ml)**	3.04±1.96	1.52±1.15	*0.155*
**Leptin (ng/ml)**	7.01±3.60	5.03±2.88	*0.705*
**PAI-1 (ng/ml)**	3.33±1.30	3.06±1.17	*0.32*
**TNF-α (pg/ml)**	21.60±14.45	13.62±10.56	*0.004*
**IL-6 (pg/ml)**	3.85±1.35	2.43±1.21	*0.012*
**IP-10 (ng/ml)**	0.47±0.18	0.30±0.19	*0.097*
**RANTES (ng/ml)**	1.75±0.67	1.62±0.63	*0.98*
**TBARS (μM)**	1.46±0.30	0.97±0.23	*0.004*

*Data are presented as mean ± SD. Paired t-test was used to compare differences in obese before and after physical exercise.*

### Correlation analysis of DNAJB3 with physical and clinical profiles, inflammatory and metabolic stress markers

To understand the physiological consequence of the reduction of DNAJB3 in obese subjects on the clinical profile as well as the inflammatory and metabolic stress responses, we investigated their correlation with DNAJB3 mRNA levels using Spearman's rank test before and after exercise. Table 5 shows that before exercise, there was a negative correlation between levels of DNAJB3 and the indicators of obesity such as BMI (r^2^ = −0.71; *P<0.0001*) and PBF (r^2^ = −0.66; *P = 0.0001*). There was also a negative correlation with TG (r^2^ = −0.36; *P<0.035*) as well as with the pro-inflammatory chemokines IP-10 and RANTES (r^2^ = −0.37; *P<0.036* and r^2^ = −0.40; *P = 0.02,* respectively). No correlation was found for the other parameters (Table 5 and data not shown). After exercise, the increased expression of DNAJB3 mRNA in obese correlated negatively with the PBF (r^2^ = −0.53; *P = 0.044*) and positively with RANTES (r^2^ = 0.75; *P<0.008*) (Table 5).

**Table 5:** Correlation between DNAJB3 mRNA expression and physical, clinical and biochemical parameters.

	Before exercise		After exercise	
	R^2^	*P-value*	R^2^	*P-value*
**BMI**	−0.709	*<0.0001*	−*0.128*	*0.650*
**PBF**	−0.656	*0.0001*	−*0.526*	*0.044*
**SLM**	−0.296	*0.12*	*0.378*	*0.165*
**HR**	−0.02	*0.945*	−*0.119*	*0.727*
**SBP**	0.038	*0.894*	*0.136*	*0.690*
**DBP**	0.136	*0.629*	*0.391*	*0.235*
**V_O2 Max_**	0.556	*0.031*	*0.220*	*0.515*
**HDL**	0.301	*0.07*	−*0.193*	*0.594*
**TG**	−0.358	*0.035*	−*0.407*	*0.214*
**Leptin**	−0.322	*0.064*	−*0.014*	*0.965*
**PAI-1**	−0.162	*0.362*	*0.277*	*0.383*
**IP10**	−0.367	*0.036*	*0.259*	*0.416*
**RANTES**	−0.404	*0.020*	*0.747*	*0.008*

*The correlation was based on ΔΔCT method and it was done on non-diabetic participants before exercise consisting of lean (n = 14), obese before exercise (n = 21) and obese after exercise (n = 17). Correlation was assessed by using Spearman's rank correlation coefficient.*

### DNAJB3 binds to JNK and IKKβ stress kinases

**Figure 4:**
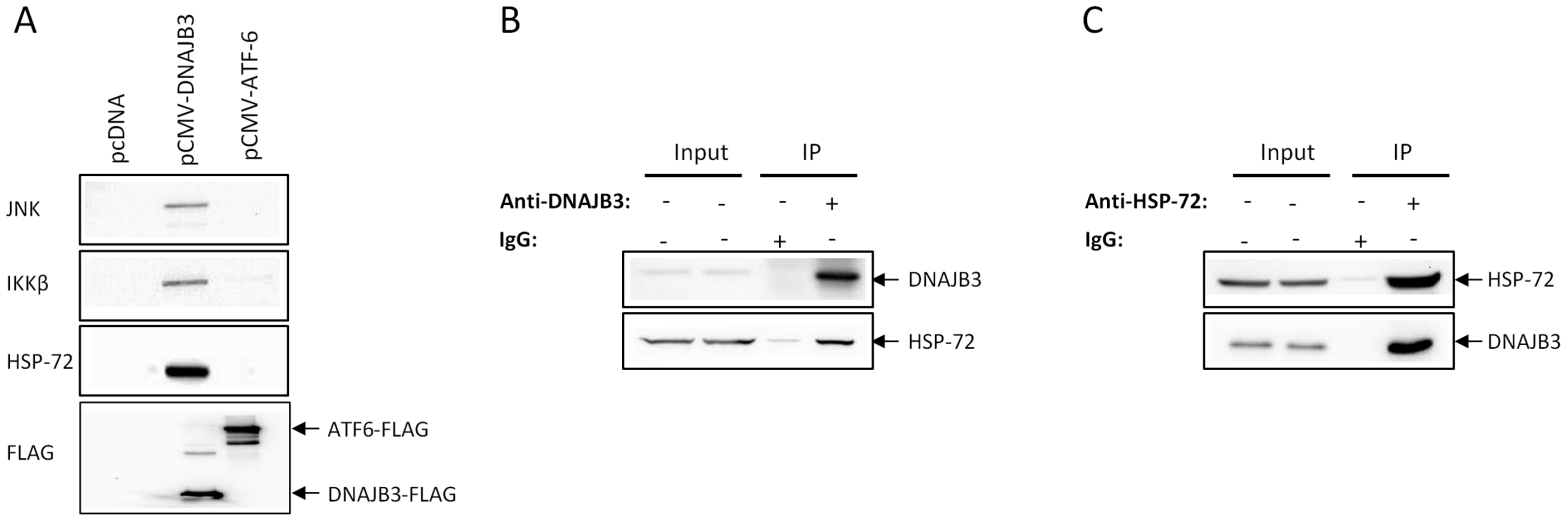
DNAJB3 forms a complex with HSP-72 and stress kinases *in vitro* by coimmunoprecipitation. (A) HEK-293 cells transfected Flag-tagged DNAJB3 and proteins lysates were coimmunprecipitated with anti-Flag antibody. Eluted proteins were subjected to western blot analysis using JNK, IKKβ and HSP-72 antibodies. Flag-tagged ATF-6 vector and pcDNA empty vector were run in parallel and used as controls. Anti-Flag antibody was also used to monitor for transfection efficiency and binding of the recombinant proteins to the anti-Flag antibody. To investigate endogenous formation of DNAJB3/HSP-72/JNK complex, whole cell lysate proteins were prepared from HEK293 cells and used to pull down the immunocomplex using either anti-DNAJB3 (B) or anti-HSP-72 (C) antibody. Eluted proteins were separated on SDS-PAGE and subjected to Western blotting using the appropriate antibodies as indicated.

In order to complement the *in vivo* data shown above, we undertook a series of *in vitro* experiments using cell lines. Based on the inverse correlation between the levels of DNAJB3 and activated JNK (Fig. 3B and 3C) and given the importance of stress kinases such as JNK, IKKβ in obesity and insulin resistance, we initially sought to determine if there is an interaction between DNAJB3 and these stress kinases. For this purpose, HEK-293 cells were transfected with pCMV-DNAJB3 and investigated the partners of interaction that might bind to DNAJB3 by coimmunoprecipitation as described in materials and methods. As negative controls, we transfected cells with pCMV-ATF-6 and pcDNA3.1 mock vector. As shown in Figure 4, we were able to detect the presence of JNK and IKKβ bands in the immunocomplex prepared from cells transfected with DNAJB3 clone. Under the same conditions, these bands were not detected in lysates prepared from cells transfected with either ATF-6 clone or with the empty vector and thus, demonstrating the specificity of the interactions. To rule out the possibility of differences in transfection efficiency between clones and/or binding affinity of the recombinant proteins to the anti-FLAG conjugated beads, we probed the membranes with anti-FLAG antibody and found that both DNAJB3 and ATF-6 clones are adequately expressed in transfected cells and they bind equally to the anti-FLAG beads (Fig. 4). Given that HSP-72 was shown in previous studies to bind and inactivate JNK and IKKβ and taking into consideration the cochaperone role of DNAJB3, we postulated that HSP-72 might be part of the coimmunoprecipated complex. Probing the membranes with anti-HSP-72 antibody revealed indeed the presence of HSP-72 in complex obtained from cell transfected with DNAJB3 clone but not from ATF-6 clone or the control vector (Fig. 4A). Our findings prompted us to investigate whether endogenous DNAJB3 could form a complex with JNK/HSP-72 by immunoprecipitation using untransfected cells using either anti-DNAJB3 or anti-HSP-72 antibody. While the interaction of JNK with either DNAJB3 or HSP-72 was inconclusive (data not shown), we were able to confirm the interaction between DNAJB3 and HSP-72 using either anti-DNAJB3 (Fig. 4B) or anti-HSP-72 (Fig. 4C) to pull down the immunocomplex.

### DNAJB3 expression is reduced *in vitro* upon activation of the ER stress

**Figure 5:**
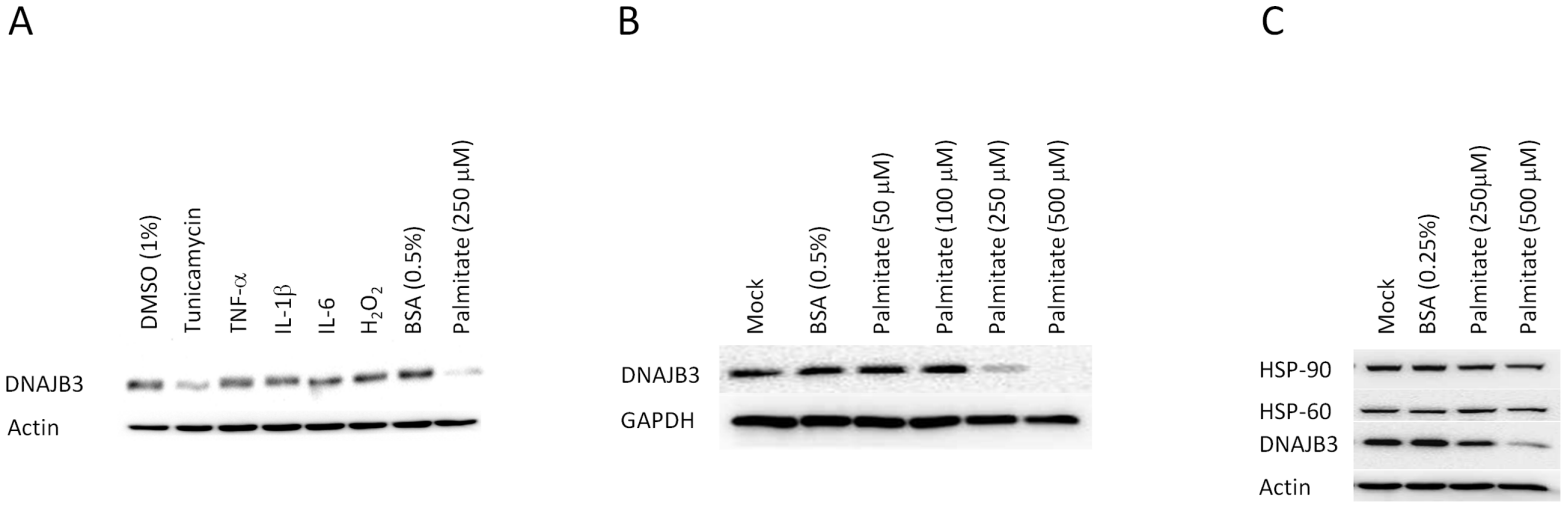
Downregulation of DNAJB protein *in vitro* following activation of ER stress by palmitate and tunicamycin. Western blot analysis using protein lysates from THP-1 cells (A) and L6 cells (B) after stimulation with 25 ng/ml of TNF-α, IL-1β and IL-6, 30 μM of H_2_O_2_, 1 μg/ml of tunicamycin and from 50 to 500 μM of palimate for overnight. DMSO at 1% and 0.5% BSA were used as controls for vehicles. Actin and GAPDH were used as internal controls to monitor for loading efficiency. (C) Effect of plamitate on the expression of HSP-60 and HSP-90 along with DNAJB3. The blots shown are representatives of at least three independent experiments with consistent results.

Low grade chronic metabolic inflammation, hyperlipidemia, and enhanced oxidative and endoplasmic reticulum (ER) stress responses are cardinal features that lead to obesity and its further progression to insulin resistance and T2D. In the context of obesity, no previous study reported the existence of mediators that could positively or negatively modulate the expression of DNAJB3. To gain new insight into the molecular mechanisms involved in regulating the expression of DNAJB3 *in vitro* using cell lines, we stimulated THP-1 and L6 cells with an array of mediators that elicit inflammation, oxidative stress and ER stress. To this end, cells were stimulated with classic inflammatory cytokines such as IL-1β, IL-6 and TNF-α, H_2_O_2_ to elicit oxidative stress, and palmitate and tunicamycin; both of them are potent inducers of the ER stress [41], [42]. As shown in Figure 5A, neither inflammatory cytokines nor H_2_O_2_ had an effect on the expression of DNAJB3 in THP-1 cells. By contrast, treatment of cells with palmitate resulted in a reduction of DNAJB3 protein in both THP-1 (Fig. 5A) and L6 (Fig. 5B) cells, while it had no effect on HSP-60 and HSP-90 proteins (Fig. 5C). In an attempt to elucidate whether the effect of palmitate on the expression of DNAJB3 was due to the activation of the ER stress or through a different pathway, we stimulated cells with tunicamycin; a chemical drug that specifically activate the ER stress. As shown in Figure 5A, tunicamycin triggers also a reduction of DNAJB3 protein. The observed downregulation of DNAJB3 following activation of ER stress by tunicamycin and palmitate is consistent with the previous study that showed a link between activation of ER stress and downregulation of DNAJB3 gene expression upon stimulation of cardiomyocytes with doxazosin [43].

## Discussion

The current study was designed to identify unexplored components of the heat shock response that might be aberrantly expressed in obese subjects and playing a possible role in the pathophysiology of obesity and insulin resistance. By complementing the *in vivo* works with the *in vitro* studies, our main findings are: 1) the expression of DNAJB3 was significantly reduced in obese subjects and physical exercise restored its normal expression; 2) DNAJB3 formed a complex with JNK, IKKβ and HSP-72 and; 3) a clear association between activation of the ER stress and reduction of DNAJB3 protein *in vitro*. The observed decrease in the expression of DNAJB3 in obese subjects was independent of the gender effect as both males and females showed similar reduction of DNAJB3 mRNA levels (data not shown). Our data demonstrating for the first time that obesity triggers a reduction in the expression of a co-chaperone at both mRNA and protein levels in human subjects add further evidence that impairment of the heat shock response is one of the key steps that lead to the development of various metabolic disorders associated with obesity.

Human DNAJB3; also known as MSJ-1 in mouse [44], is one of the 49 members of the HSP-40 family of proteins and it acts as a co-chaperone to regulate the activity and specificity of HSP-70; a major component of the proteostasis network [27], [45], [46]. DNAJB members exert their role by stimulating the ATPase activity of HSP-70 through their J-domain, thereby keeping the bound substrates in successive refolding cycles [47], [48]. They are also known for their ability to deliver a diverse set of substrates to HSP-70 and thus, determining substrate specificity [46], [49]. Given their dual mode of action, DNAJB-type proteins are classified among the strongest protectors against protein toxicity associated with protein aggregation [50], [51]. This makes DNAJB family of proteins interesting targets for therapy against protein folding diseases either through functional modulation of their activity or by increasing their expression.

There is a widespread clinical interest in the protective role of HSPs against a variety of diseases, including obesity, insulin resistance and diabetes. Attenuation of this important host defense system is associated with various clinical manifestations and pathological disorders. The findings of our current investigation confirm the previous gene expression profiling study in which DNAJB3 was among the list of genes downregulated in obese mice compared to lean mice [40]. In our model, the significant decrease of DNAJB3 in obese subjects, its correlation with inflammatory markers and fat levels and the restoration of its normal expression after a defined exercise protocol suggest that DNAJB3 might potentially play a protective role in obesity, insulin resistance and T2D. In support of this, the decrease of DNAJB3 was more pronounced in diabetic than in non-diabetics subjects (data not shown). Recently, it was proposed that T2D is the result of a metabolic paradigm in which metabolic inflammation, insulin resistance and impairment of the HSR work in a vicious cycle [11]. Obesity, sedentary lifestyle and high fat calorie perpetuate this cycle by lowering HSPs and thus, leading to metabolic inflammation and impairment of insulin signaling [5], [16], [29]. The downregulation of DNAJB3 in clinically relevant tissue organ can be added to the list of component of the HSR that are attenuated by obesity in human subjects. Our data illustrate also the complexity of the HSR to protect from metabolic disorders associated with obesity. The previous studies that investigated the status of the HSR in the context of obesity, insulin resistance and diabetes were carried out on the skeletal muscle of T2D patients and they showed a reduced expression of HSP-72 that correlates with the degree of insulin resistance [8], [9], [33]. The findings in human subjects were further supported in experimental animal models demonstrating impaired expression of HSP-72 in the rat model of streptozotocin-induced diabetes [32] and reduced expression of both HSP-25 and HSP-72 in the insulin-resistant aged rats [52], [53]. As a note of caution, our data did not explain the exact significance of this reduction to obesity and this may represent a limitation of this study, nonetheless, further studies that are beyond the scope of this work such as using DNAJB3 knockout mouse animal models as well as treatment with pharmacological modulators of DNAJB3 are warranted.

Beside their chaperone activity, HSPs are well known for their anti-inflammatory and anti-stress properties by binding to JNK and IKKβ stress kinases and concomitantly suppressing their activities [11], [54]. In order to gain new insights into the role that DNAJB3 may play in the context of obesity and the functional consequences associated with its reduction in obese subjects, we sought to investigate the partners of interaction that associate with it using coimmunoprecipiation assays. Under our experimental conditions, we identified HSP-72 and two stress kinases namely JNK and IKKβ as part of the complex that specifically copurified with DNAJB3 protein (Fig. 4). Interestingly, all these partners have been linked to obesity, insulin resistance and T2D [5], [10], [16]. Our current findings raise a series of fundamental questions for future follow-up studies to elucidate the role of this understudied co-chaperone protein on metabolic diseases. One of the eminent questions is the functional consequences of these interactions on the activity of these stress kinases and if so, does DNAJB3 acts alone or in cooperation with HSP-72? Additionally, does DNAJB3 interact directly with these proteins or not? Does heat therapy induce the expression of DNAJB3 such as it is the case for HSP-72? For instance, overexpression of HSP-72 by prior heat conditioning or by ectopic expression can markedly block the activation of JNK both *in vitro* and *in vivo* [33], [55], [56] and prevent NF-κB [57]. Together, these observations illustrate the detrimental consequences associated with the activation of JNK and IKKβ stress kinases in key metabolic sites when the HSR is blunted.

Since physical exercise was shown to exert favorable effects on obesity, insulin resistance and diabetes; at least in part due to the induction of heat shock response [58], we speculated whether it can restore the expression of DNAJB3 in obese subjects with concomitant improvement of metabolic stress and clinical outcomes. As expected, our regular exercise protocol upregulated the expression of DNAJB3 and also reduced the expression of phosphoryated JNK (Fig. 3). While the negative effect of exercise on JNK phosphorylation is well established both in human and animals models [59], the upregulation of DNAJB3 by physical exercise is novel. In our case, the effect of exercise on the increase of DNAJB3 expression was at the mRNA and protein levels and it was observed in both PBMC and subcutaneous adipose tissue. Our results are similar to those reported for HSP-72 in which they showed that all the interventions that lead to the induction of HSP-72 expression; including exercise are associated with impairment of JNK phosphorylation with concomitant improvement of clinical outcomes in humans and animal models of obesity, insulin resistance and T2D [52], [58], [59], [60], [61]. Likewise, activated HSP-25 was shown to bind to IKKβ and inhibits its activity and thereby, improving insulin signaling in skeletal muscle from high fat diet-fed rats [36], [37], [56], [62]. In the present study, it is unclear whether there is a direct role of DNAJB3 on JNK and IKKβ activities or not, but from our immunopreciptation studies, the fact that DNAJB3 is part of a complex that contains JNK, IKKβ along with HSP-72 suggests that DNAJB3 might reside in the pathway modulating the activity of these stress kinases.

The chronic conditions associated with obesity such as low grade metabolic inflammation, hyperlipidemia, and enhanced oxidative and ER stress responses promoted us to explore the possible mechanisms involved in modulating the expression of DNAJB3. From our *in vitro* studies, only palmitate and tunicamycin were shown to trigger a significant reduction in the expression of DNAJB3 protein (Fig. 5). Under the same conditions, the expression of HSP-60 and HSP-90 were not affected by any of these treatments. Palmitate is a saturated free fatty acid well known for its cytotoxic effect. In addition to its ability to induce ER stress, palmitate acts also by increasing the levels of ceramide, reactive oxygen and nitric oxygen species, alteration of mitochondrial function [41], [42]. The observed inhibition of DNAJB3 expression with palmitate and its confirmation with tunicamycin suggest that the ER stress is involved in the downregulation of DNAJB3. Our observation is in agreement with a previous study showing more than 5-fold decrease in the expression of DNAJB3 mRNA following stimulation of HL-1 cardiomyocytes with doxazosin, a potent inducer of the ER stress [43]. In their study, the authors also detected the increased expression of two ER stress related transcription factors, namely CAAT enhancer binding protein β (C/EBPβ) and growth arrest and DNA-damage inducible protein 153 (GADD153) also known as C/EBP-homologous protein (CHOP) [43]. Interestingly, a recent study showed that stress-induced expression of CHOP was associated with repression MyoD, a gene involved in muscle differentiation [63]. The murine *dnajb*3 promoter has two C/EBP binding sites [64] and their role in the regulation of DNAJB3 following activation of the ER stress, if any, remains to be demonstrated. Further clarifications are also needed to confirm whether or not the observed inhibition of DNAJB3 *in vitro* following activation of the ER stress with palmitate occurs also *in vivo* in obese subjects. In our study population, we detected high levels of CHOP as well as the spliced form of the ER stress response protein, X-box-binding protein 1 (XBP1) in obese subjects (data not shown) which indicates that the ER stress is induced in obese subjects. On the other hand, our data did not rule out the effect of other inflammatory mediators since we only selected in our study a few representatives of inflammatory cytokines.

Another limitation of the study is the fact that obese subjects that participated in this study are relatively older than lean subjects (Table 1). In this regard, aging is well known to decrease the expression of HSPs such as HSP-72 [65], [66]. However, the fact that, in one hand, the expression of HSC-70 and HSP-90 were increased in obese subjects and on the other hand, the positive effect of exercise in restoring the normal expression of DNAJB3 suggest that aging is unlikely to affect the expression of DNAJB3.

In conclusion, we provided compelling evidence that the expression of the co-chaperone DNAJB3 is markedly reduced at the mRNA and protein levels in both PBMC and adipose tissue of obese subjects. We further demonstrated that physical exercise restores the normal expression of DNAJ3 to the levels comparable to lean subjects. Although we did not demonstrate the causal relationship between reduced expression of DNAJB3 and obesity, we demonstrated that DNAJB3 is part of a complex that contains key proteins involved in obesity, insulin resistance and T2D such as HSP-72, JNK and IKKβ. All together, our data support the suggestion that DNAJB3 can potentially play a protective role against obesity, and thus targeting DNAJB3 may have a potential therapeutic benefit for the control and management of obesity and insulin resistance.
